# A Nomogram for Predicting Survival for Patients with Brain Metastatic and *EGFR* Mutation Advanced Non-Small Cell Lung Cancer

**DOI:** 10.32604/or.2024.053363

**Published:** 2025-03-19

**Authors:** JIYUN PANG, WEIGANG XIU, YUEYUN CHEN, WENJING LIAO, QIN ZHANG, HUASHAN SHI

**Affiliations:** 1 Department of Thoracic Oncology and State Key Laboratory of Biotherapy, Cancer Center, West China Hospital, Sichuan University, Chengdu, 610041, China; 2 West China School of Medicine, Sichuan University, Chengdu, 610041, China; 3 West China School of Medicine, Department of Postgraduate Students, Sichuan University, Chengdu, 610041, China; 4 Department of Biotherapy, Cancer Center, West China Hospital, Sichuan University, Chengdu, 610041, China

**Keywords:** Brain metastasis (BM), Epidermal growth factor receptor (*EGFR*), Tyrosine kinase inhibitor (TKI), Tumor microenvironment, Non-small cell lung cancer (NSCLC)

## Abstract

**Background:**

Non-small cell lung cancer (NSCLC) is often accompanied by brain metastasis (BM), and the prognosis of patients with BM is poor. This study assesses the prognostic impact of BM in NSCLC patients with epidermal growth factor receptor (*EGFR*) mutations.

**Methods:**

We retrospectively evaluated 692 advanced NSCLC patients with *EGFR* mutations treated with tyrosine kinase inhibitors (TKIs) at West China Hospital from 2015 to 2019. The overall survival rate (OS), progression-free survival rate (PFS), objective response rate (ORR), disease control rate (DCR), and clinical parameters of the BM and non-BM groups were compared. Univariable and multivariable regressions were performed to identify independent prognostic factors, followed by validation of a predictive nomogram using receiver operating characteristics and calibration curves. Immune infiltration in tumor tissues was assessed by immunostaining.

**Results:**

NSCLC patients with BM exhibited a higher frequency of other-site and multi-organ metastases than those without BM. The BM group demonstrated significantly worse OS (26.2 *vs*. 39.1 months, *p* < 0.001) and PFS (12.3 *vs*. 18.8 months, *p* < 0.001), although the DCR (*p* = 0.831) and ORR (*p* = 0.653) were similar in both groups. BM was identified as an independent predictor of poor prognosis. The nomogram performed well, achieving a C index of 0.73, with consistent calibration curves for predicted and actual prognoses. Additionally, fewer peripheral lymphocytes were observed in the BM group.

**Conclusions:**

BM is a significant risk factor for NSCLC patients, potentially linked to lymphocytopenia.

## Introduction

Lung cancer is the most prevalent malignancy worldwide and also ranks first in terms of mortality rates [1]. Non-small cell lung cancer (NSCLC) accounts for 85% of all lung tumors and is histologically classified into adenocarcinomas (LUAD), squamous cell carcinomas (LUSC), and large cell carcinomas [2]. An estimated 20%–40% of the patients in the advanced stages of cancer develop brain metastases (BM), and lung cancer alone accounts for 59% of all BM [3]. BM occurs in almost half of the lung cancer cases and significantly worsens patient prognosis [4–6]. Furthermore, epidermal growth factor receptor (*EGFR*) mutations occur in 44%–63% of NSCLC patients and are established driver oncogenes for NSCLC that can increase the risk of BM [7,8]. *EGFR*-targeting tyrosine kinase inhibitors (*EGFR*-TKIs), such as gefitinib, erlotinib, and afatinab, have shown promising results in treating advanced NSCLC [9]. Compared to cytotoxic drugs or whole brain radiotherapy (WBRT), *EGFR*-TKIs can reduce the progression risk of the central nervous system (CNS) in NSCLC patients harboring *EGFR* mutations. In addition, *EGFR*-TKIs have demonstrated significant clinical benefits in terms of progression-free survival (PFS) and objective response rate (ORR), with more tolerable side effects [10–12].

The tumor microenvironment, especially the immune landscape, is a key determinant of patient prognosis and treatment outcomes [13,14]. However, whether the intracranial lymphatic system influences the survival of cancer patients with BM is unclear. This study aimed to determine the impact of BM on the prognosis of NSCLC patients treated with *EGFR*-TKIs and elucidate the potential mechanisms from the perspective of the immune system.

## Materials and Methods

### Patients and data collection

The Hospital Information System of West China Hospital, Chengdu, China was screened for treatment-naïve patients with pathologically confirmed, *EGFR*-mutated, metastatic NSCLC, who received *EGFR*-TKI as first-line therapy. The inclusion criteria were: (1) histologically confirmed stage IV metastatic NSCLC; (2) first-line chemotherapy (*EGFR*-TKI) as the sole therapy; (3) Eastern Cooperative Oncology Group (ECOG) performance status of 0–3; and (4) with *EGFR* mutation. The Human Investigation Committee (IRB) of West China Hospital, Sichuan University approved this study (20211349). Informed written consent was obtained from all study participants. Following the criteria, 692 patients were selected and divided into the BM (n = 346) and non-BM (n = 346) groups. The median age was 58 years in the BM group and 59 years in the non-BM group. TNM staging was established according to the guidelines of the 8th edition of the American Joint Committee on Cancer/Union for International Cancer Control [15] Data on age, gender, smoking history, histological classification, progression type, therapy (including targeted therapy with or without chemotherapy, radiation, or immunotherapy), and peripheral lymphocyte counts were collected. The range of lymphocyte and cutoff values was determined based on laboratory parameters and prior reports [16–18]. The United Nations has identified 60 years as a criterion for the older population in developing countries [19,20]. PFS was defined as the time from initiating first-line therapy to confirmed disease progression or death, and overall survival (OS) was defined as the time from diagnosis to death from any cause or the date of the last follow-up. Follow-up was conducted at regular intervals until death or until December 2019 through clinical visits or telephone interviews.

### Diagnosis of BM

The diagnostic method was selected per the judgment of the attending physician. According to the current diagnostic guidelines, possible BM was detected using enhanced computed tomography (CT) scans. Enhanced magnetic resonance imaging (MRI) or ^18^F-fluorodeoxyglucose positron emission tomography (^18^F-FDG PET) imaging was performed in cases where CT scans were insufficient to determine the presence of BM. All diagnoses were verified by two or more oncologists and imaging specialists, and the diagnostic guidelines were consistent during the observation period.

### Statistical analysis

**Figure 1:**
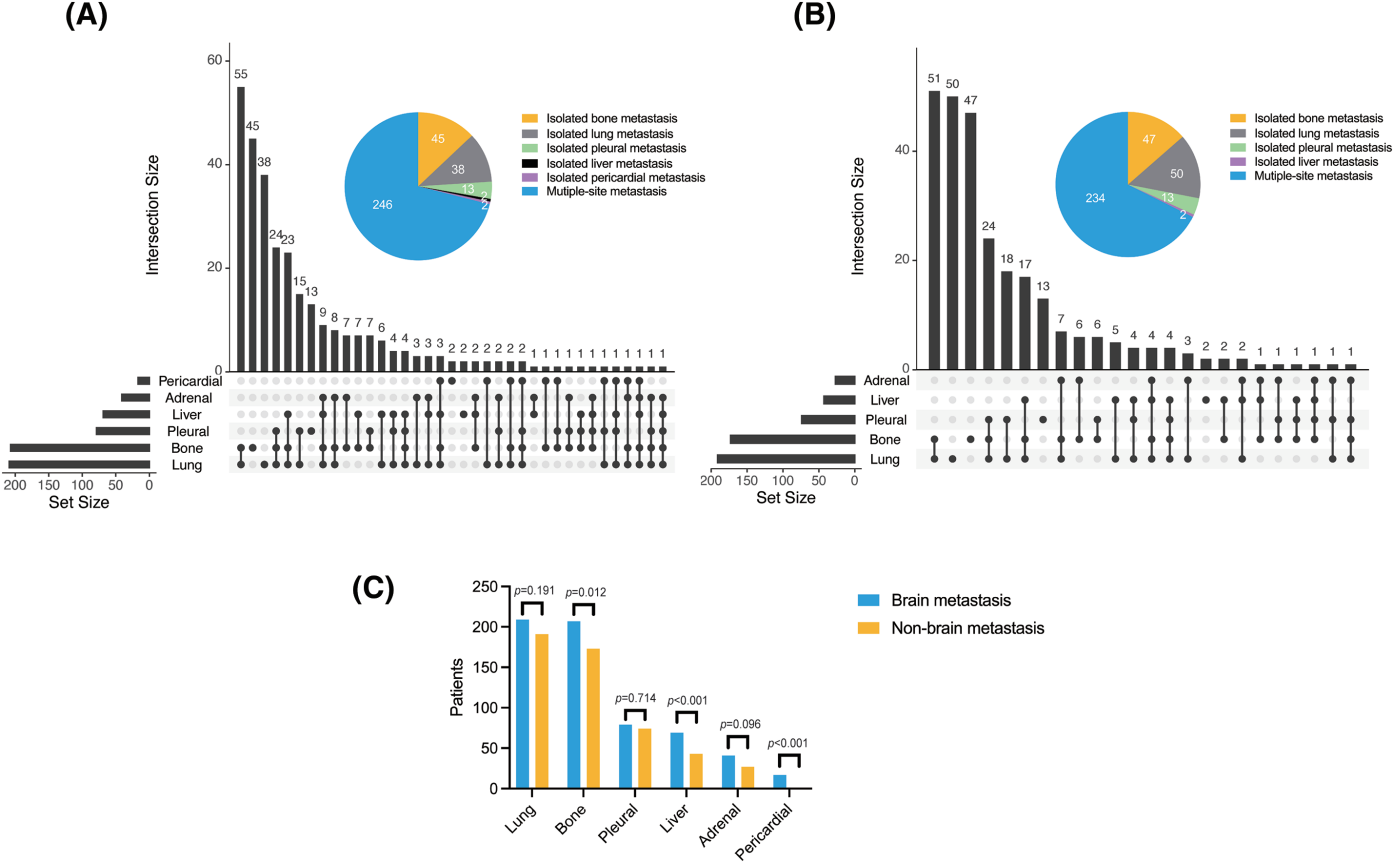
Organ metastases in the (A) BM and (B) non-BM groups, and (C) comparison of both groups

**Figure 2:**
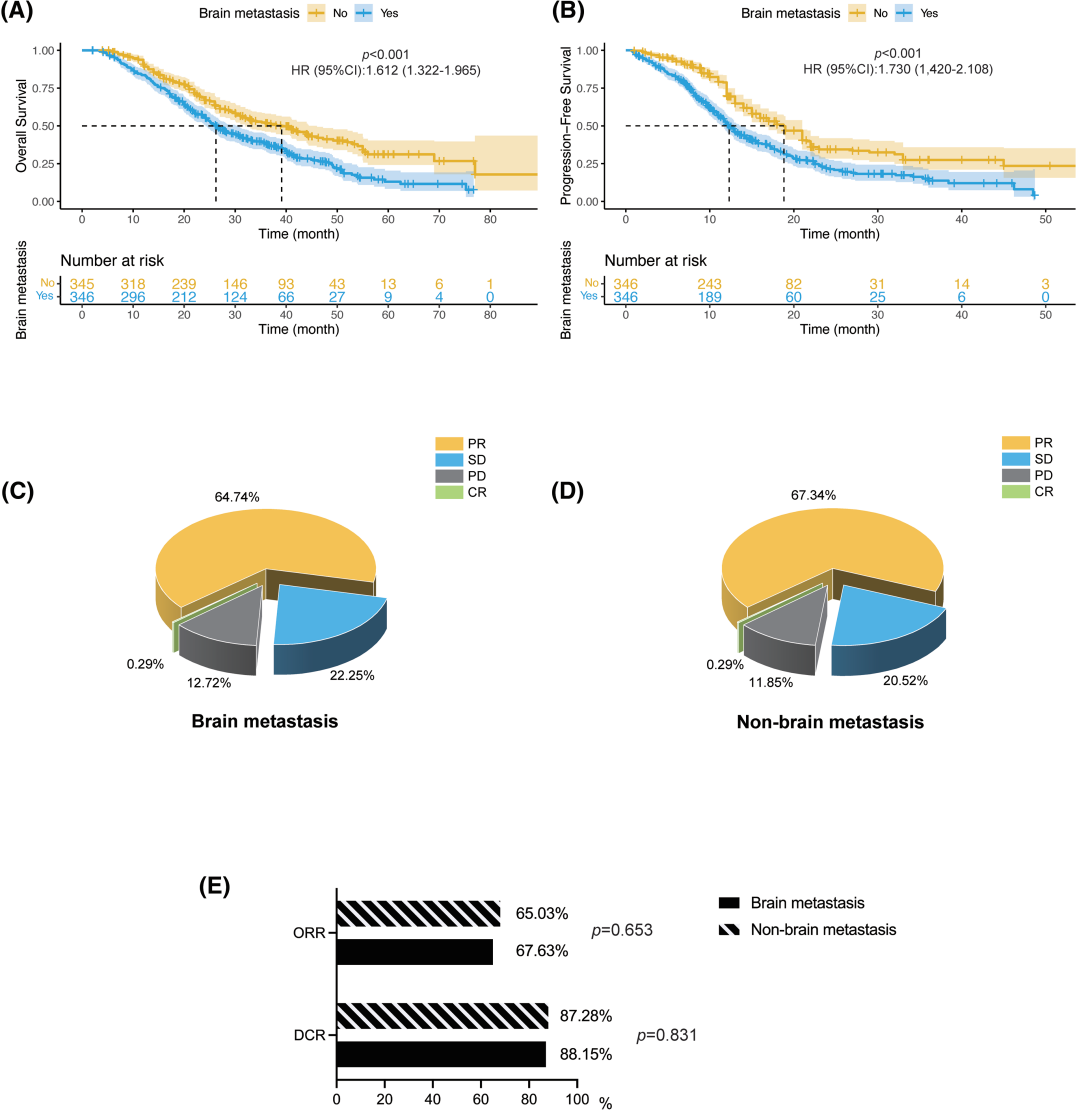
BM affects the survival and treatment outcomes for NSCLC patients. (A and B) The OS and PFS of NSCLC patients in the (A) BM and (B) non-BM groups after *EGFR*-TKIs therapy. PR/CR in the (C) BM and (D) non-BM groups. (E) Column chart showing ORR in the BM and non-BM groups. HR, hazard ratio; PR, partial response; SD, stable disease; PD, progressive disease; CR, complete response.

**Figure 3:**
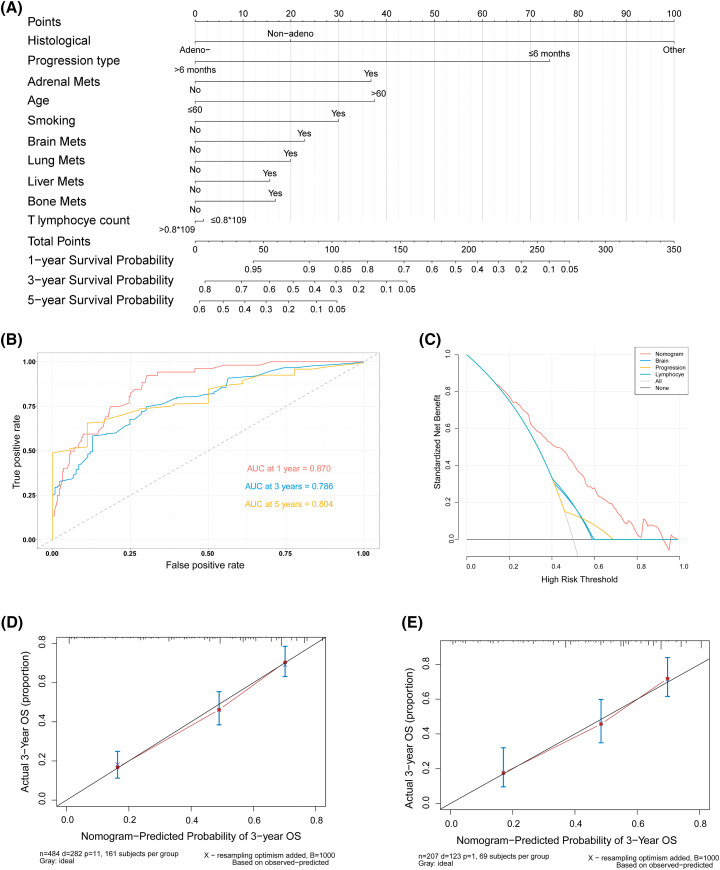
A nomogram and the validation for predicting the OS of patients. (A) Nomogram for predicting 1-, 3-, and 5-year OS of 484 patients (7/10 of the total number) obtained by random sampling. (B) ROC curves for 1-, 3-, and 5-year OS in the training cohort. (C) DCA for the nomogram and three other factors. (D) Calibration curves for internal validation to predict 3-year OS in the training cohort. (E) Calibration curves for external validation of 3-year OS with 3/10 of the total number of patients (n = 208) obtained by random sampling. AUC, the area under the curve; DCA, decision curve analysis; ROC, receiver operating characteristic

Statistical analyses were conducted using SPSS version 26.0 (IBM Corp., Armonk, NY, USA) and R (R packages, version 4.2.2). The following R packages were used: survival (version 3.2-13) for survival analysis, UpSetR (version 1.4.0) for Fig. 1, survminer (version 0.4.9) for Fig. 2, MatrixModels (version 0.5-0), haven (version 2.5.1), stringi (version 1.7.6), Hmisc (version 5.1-1), lattice (version 0.20-45), Formula (version 1.2-5), ggplot2 (version 3.4.4), foreign (version 0.8-82), rms (version 6.5-0) and timeROC (version 0.4) for Fig. 3 [16,21]. According to Cochran’s rule, the Chi-square test or Fischer’s exact test was employed to compare qualitative variables. PFS and OS curves for the BM and non-BM groups were plotted using the Kaplan-Meier method, and survival rates were compared using the log-rank test. Independent predictive factors of BM were further identified through multivariate analysis using a Cox proportional hazards model. Factors demonstrating significant association in the univariate analysis (*p* < 0.05) were included in the multivariate analysis. The independent variables were incorporated into the Cox regression model using the enter method. The independent risk factors were included in a prognostic nomogram. Receiver operating characteristic (ROC) analysis, decision curve analysis (DCA), and calibration curves were plotted to evaluate the nomogram’s predictive accuracy, clinical applicability, and discriminative ability. *p-*values < 0.05 were deemed statistically significant.

## Results

### Baseline characteristics

A total of 692 patients were included in this study, comprising 274 (39.6%) males and 418 (60.4%) females. The mean age of the participants was 58.4 years, ranging from 22 to 83 years. All patients were categorized into the BM (n = 346) and non-BM (n = 346) groups. Gender distribution, smoking history, *EGFR* mutation, and other clinical parameters were similar in both groups. Patients with BM tended to be younger (median age 58 years) than those in the non-BM group (median age 59 years), albeit without statistical significance. *EGFR* mutations included *exon 19* deletion (n = 350 [50.6%]), *L858* substitution (n = 297 [42.9%]), and others (n = 45 [6.5%]). Additionally, 666 (96.2%) of the patients were diagnosed with adenocarcinomas, among whom 50% had BM and the remaining 50% did not exhibit BM (non-BM group). Patients received targeted therapy alone (n = 471 [68.1%]) or in combination with chemotherapy, radiation, or immunotherapy (n = 221 [31.9%], Table 1).

**Table 1:** Clinical characteristics of NSCLC patients receiving *EGFR*-TKI treatment

Characteristic	Brain metastasis	Non-brain metastasis	*p*-value
**Total N (%)**	346 (50.0%)	346 (50.0%)	
**Gender**			
**Female**	208 (49.8%)	210 (50.2%)	
**Male**	138 (50.4%)	136 (49.6%)	1.000
**Age group**			
**<60 years**	184 (51.5%)	173 (48.5%)	
**≥60 years**	162 (48.4%)	173 (51.6%)	0.447
**Smoking history**			
**Yes**	80 (48.8%)	84 (51.2%)	
**No**	266 (50.4%)	262 (49.6%)	0.789
**EGFR mutation**			
***Exon 19* deletion**	178 (50.9%)	172 (49.1%)	
***L858R* mutation**	146 (49.2%)	151 (50.8%)	
**Other**	22 (48.9%)	23 (51.1%)	0.901
**Histological type**			
**Adenocarcinoma**	333 (50.0%)	333 (50.0%)	
**Non-adenocarcinoma**	13 (50.0%)	13(50.0%)	1.000
**Combination therapies**			
**Yes**	115 (52.0%)	106 (48.0%)	
**No**	231 (49.0%)	240 (51.0%)	0.166
**Progression type**			
**Fast (≤6 months)**	69 (53.5%)	60 (46.5%)	
**Slow (>6 months)**	277 (49.2%)	286 (50.8%)	0.380
**T lymphocyte number**			
**>0.8 × 10^9^**	41 (12.6%)	284 (87.4%)	
**<0.8 × 10^9^**	305 (83.1%)	62 (16.9%)	0.452

Note: NSCLC, non-small cell lung cancer; HR, hazard ratio; *EGFR*, epidermal growth factor receptor.

### Organ metastasis

The lungs (60.4%), bone (59.8%), pleura (22.8%), liver (19.9%), adrenal glands (11.8%), and pericardium (4.9%) were most frequently colonized in the BM group. Lung metastases were defined as significantly enhanced bilateral lung lesions in contrast-enhanced CT images. And tumor lesions beyond the unilateral lung and regional node were determined as distant metastases. Thoracic radiologists independently reviewed these images to diagnose lung metastases. Notably, lung cancer can spread directly through the airways, facilitating metastasis within the lungs. In the non-BM group, metastasis was detected in the lungs (55.2%), bones (50%), pleura (21.4%), liver (12.4%), and adrenal glands (7.8%), but not in the pericardium. Bone (*p* = 0.012), liver (*p* < 0.001), and pericardial (*p* < 0.001) metastases were more common in the BM group, and the frequencies of adrenal (*p* = 0.096), lung (*p* = 0.191), and pleural (*p* = 0.714) metastases were slightly higher in the BM group compared to the non-BM group, albeit without statistical significance. In addition, the frequency of multiple organ metastasis (≥3) in the BM group was 27.7% (96/346) compared to only 19.7% (68/346) in the non-BM group (*p* < 0.001; Fig. 1). Thus, BM in NSCLC patients increase the likelihood of metastases to other organs.

### Survival analysis

The median follow-up times for the BM and non-BM groups were 42.1 months (95% confidence interval (CI): 37.1–47.1 months) and 40.3 months (95% CI: 38.0–42.6 months), respectively. The BM group had a significantly shorter median OS of 26.2 months (95% CI: 24.2–30.4 months) compared to 39.1 months (95% CI: 32–46 months) in the non-BM group (hazard ratio (HR) = 1.612, 95% CI: 1.322–1.965, *p* < 0.001). Likewise, the median PFS was only 12.3 months (95% CI: 11.5–13.6 months) in the BM group compared to 18.8 months (95% CI: 16–21 months) in the non-BM group (HR = 1.73, 95% CI: 1.420–2.108), *p* < 0.001; Fig. 2A,B). One patient in each BM group and the non-BM group achieved CR (Fig. 2C,D). The DCR was similar for both groups at 87.28% and 88.15%, respectively (*p* = 0.653), as was the ORR at 65.03% and 67.63%, respectively (*p* = 0.831) (Fig. 2E).

### Univariate and multivariate Cox regression

Univariate analysis revealed that factors such as advanced age, gender, absence of exon deletion, *L858R* mutation, non-adenocarcinoma histological type, smoking, rapid progression, reduced T lymphocyte counts in the tumor, and metastases to the lung, liver, adrenal gland, bone, and brain were associated with poor OS and PFS. Variables showing significant associations with prognosis (*p* < 0.05) in the univariate analysis were included in the multivariate analyses. The multivariate analysis further identified age, histological type, smoking, type of progression, number of T lymphocytes, and metastases to the lung and distant sites as independent predictors of OS (Table 2). Additionally, age, histological type, type of progression, smoking, and metastases to the adrenal glands, bone, and brain were determined to be independent predictors of PFS (Table 3).

**Table 2:** Factors influencing OS of NSCLC patients (n = 692)

Variable	Univariate analysis		Multivariate analysis	
	HR (95%CI)	*p*-value	HR (95%CI)	*p*-value
Age		<0.001		<0.001
<60 *vs*. ≥60 years	1.027 (1.017–1.036)		1.030 (1.020–1.041)	
Gender		0.001		0.310
Male *vs*. Female	0.724 (0.595–0.881)		0.875 (0.676–1.133)	
Histological classification		<0.001		<0.001
Adenocarcinoma *vs*. Non-adenocarcinoma	1.986 (1.481–2.662)		2.114 (1.565–2.856)	
Smoking history		<0.001		0.002
No *vs*. Yes	1.727 (1.387-2.150)		1.575 (1.177–2.107)	
*Exon 19* deletion		0.001		0.432
No *vs*. Yes	0.722 (0.594–0.878)		0.841 (0.545-1.297)	
*L858R* mutation		<0.001		0.616
No *vs*. Yes	1.411 (1.161–1.715)		1.118 (0.723–1.728)	
Other *EGFR* mutation		0.788		0.717
No *vs*. Yes	0.945 (0.625–1.428)		1.088 (0.672–1.233)	
Combination therapies		0.367		0.414
No *vs*. Yes	0.908 (0.736–1.121)		—	
Progression type		<0.001		<0.001
≤6 *vs*. >6 months	0.534 (0.422–0.677)		0.487 (0.381–0.623)	
T lymphocyte number		<0.001		<0.001
<0.8 *vs*. ≥0.8 × 10^9^	0.637 (0.522–0.778)		0.791 (0.604–1.036)	
Lung metastasis		<0.001		0.004
No *vs*. Yes	1.521 (1.242–1.862)		1.359 (1.101–1.676)	
Liver metastasis		<0.001		0.018
No *vs*. Yes	1.985 (1.565–2.518)		1.359 (1.052–1.755)	
Adrenal gland metastasis		<0.001		<0.001
No *vs*. Yes	2.667 (2.014–3.530)		2.162 (1.616–2.892)	
Bone metastasis		<0.001		<0.001
No *vs*. Yes	1.873 (1.526–2.297)		1.500 (1.211–1.857)	
Brain metastasis		<0.001		0.009
No *vs*. Yes	1.612 (1.322–1.965)		1.426 (1.093–1.861)	

Note: NSCLC, non-small cell lung cancer; HR, hazard ratio; *EGFR*, epidermal growth factor receptor.

**Table 3:** Factors influencing PFS of NSCLC patients (n = 692)

Variable	Univariate analysis		Multivariate analysis	
	HR (95% CI)	*p*-value	HR (95% CI)	*p*-value
Age		<0.001		<0.001
<60 *vs*. ≥60 years	1.017 (1.007–1.026)		1.017 (1.008–1.027)	
Gender		0.018		0.525
Male *vs*. Female	0.790 (0.649–0.961)		0.919 (0.708–1.193)	
Histological classification		0.01		0.011
Adenocarcinoma *vs*. Non-adenocarcinoma	1.443 (1.091–1.909)		1.459 (1.091–1.952)	
Smoking history		<0.001		<0.001
No *vs*. Yes	1.695 (1.363–2.109)		1.770 (1.325–2.364)	
*Exon 19* deletion		<0.001		0.235
No *vs*. Yes	0.636 (0.523–0.775)		0.764 (0.490–1.191)	
*L858R* mutation		<0.001		0.336
No *vs*. Yes	1.625 (1.336–1.978)		1.246 (0.796–1.949)	
Other *EGFR* mutation		0.633		0.442
No *vs*. Yes	0.912 (0.604–1.379)		0.932(0.496–1.051)	
Combination therapies		0.759		
No *vs*. Yes	1.033 (0.838–1.274)		0.377 (0.242–0.866)	
Progression type		<0.001		<0.001
≤6 *vs*. >6 months	0.423 (0.333–0.536)		0.287 (0.219–0.376)	
T lymphocyte number		<0.001		0.954
<0.8 *vs*. ≥0.8 × 10^9^	0.627 (0.513–0.765)		0.992 (0.759–1.297)	
Lung metastasis		<0.001		<0.001
No *vs*. Yes	1.492 (1.219–1.825)		1.466 (1.186–1.813)	
Liver metastasis		<0.001		0.046
No *vs*. Yes	1.739 (1.373–2.203)		1.108 (0.852–1.441)	
Adrenal gland metastasis		<0.001		<0.001
No *vs*. Yes	2.353 (1.780–3.111)		1.778 (1.330–2.378)	
Bone metastasis		<0.001		<0.001
No *vs*. Yes	1.875 (1.529–2.299)		1.660 (1.336–2.061)	
Brain metastasis		<0.001		<0.001
No *vs*. Yes	1.730 (1.420–2.108)		2.255 (1.703–2.985)	

Note: NSCLC, non-small cell lung cancer; HR, hazard ratio; *EGFR*, epidermal growth factor receptor.

### Nomogram development and validation

Based on the results above, a predictive nomogram for OS was developed using age, gender, histological classification, smoking history, progression type, T lymphocyte number, and multi-organ metastases as the independent risk factors (Fig. 3A). 484 patients were included as the training cohort, obtained by random sampling. The total scores for predicting 1-, 3-, and 5-year OS were calculated by adding that of the individual predictive factors. The nomogram exhibited good discriminatory ability, as evidenced by the area under the ROC curves and a C-index of 0.73. The area under the curve summarized the overall diagnostic accuracy of the test (Fig. 3B), while the DCA indicated its clinical practicality (Fig. 3C) [22]. Furthermore, the actual and predicted 3-year OS rates were proven to be concordant with internal validation (484 patients) and external validation (the remaining 208 patients) (Fig. 3D–E).

## Discussion

Over 90% of cancer-related deaths are attributed to metastasis, and this process is regulated by multiple factors related to the tumor microenvironment [23,24]. Lung cancer cells preferentially metastasize to the brain, with the highest frequency of BM observed in small cell lung cancer (SCLC) at 31%, followed by LUADs at 21%, large cell carcinomas at 21%, and LUSCs at 8% [4,25]. Several hypotheses, starting from the “seed and soil” theory proposed by Paget in 1889, have been put forth to explain the mechanisms underlying tumor metastasis, including epithelial-mesenchymal transition (EMT), cancer stem cells, and circulating tumor cells (CTCs) [26]. Several studies have shown that BM negatively impacts the prognosis of lung cancer. While *EGFR*-TKIs have achieved higher efficacy than other treatment modalities, the impact of BM on the prognosis of NSCLC patients following *EGFR*-TKI therapy remains poorly understood.

Advanced age can significantly worsen both the overall and progression-free survival of lung cancer patients, which can be attributed to the age-related decline in immune function and poor tolerance to treatment [27]. Furthermore, adenocarcinomas have a better prognosis than non-adenocarcinomas, most likely due to the biological characteristics of LUADs and recent therapeutic advances [28]. Smoking has been associated with a significant increase in the risk of lung cancer [29]. There is evidence that smoking induces the aggregation of the M2-type tumor-associated macrophages (TAMs) around NSCLC tissues and accelerates tumor development [30]. In addition, patients with multi-organ metastases had poor OS and PFS, which may be associated with higher tumor load, more complications due to invasion of multiple organs, and poor treatment effect. Rapid tumor progression generally indicates a worse prognosis due to primary drug resistance, which enhances the proliferative capacity and invasiveness of tumor cells, leading to suboptimal treatment effects. Furthermore, secondary mutations in *KRAS*, *C-MET*, and *TP53* [30] contribute to tumor heterogeneity and unpredictable cancer development, increasing malignancy and adversely affecting patient survival. Increasing evidence also suggests that BM negatively impacts the outcomes of *EGFR*-TKI therapy in NSCLC patients [31–33]. This effect could be attributed to the distinct genetic landscapes of the primary tumor and the metastatic clones, as well as the low concentration of the drug in cerebrospinal fluid.

Interestingly, the peripheral lymphocyte counts were markedly lower in the patients with BM than in the non-BM group in this study. According to the univariate Cox regression model, peripheral lymphocyte count <0.8 × 10^9^/L correlated with worse OS, which underscores the impact of the tumor microenvironment on treatment outcomes and prognosis. Results from other studies have also indicated that the number of peripheral blood lymphocytes is negatively correlated with the survival of cancer patients [34,35]. Ikarashi et al. [36] reported lower infiltration and abundance of various immune cell populations in the BM microenvironment, including CD4+ T cells, CD8+ T cells, and Tregs. These novel insights regarding the tumor immune microenvironment could be crucial in optimizing therapeutic strategies by modulating immune functions and improving OS for NSCLC patients.

This study has certain limitations that should be addressed. First, there were inevitable biases due to its retrospective design. In addition, there may have been selection bias since the treatment was determined at the attending physician’s discretion rather than randomization. This is relevant as the radiological imaging tests used to detect BM differ in specificities and sensitivities. Furthermore, the sample size in the immunofluorescence experiments was small, which limits the generalization of the results. Finally, this was a single-center study, and the findings must be validated using multi-center cohorts.

BM often deteriorates the quality of life and imposes a significant financial burden on patients. Consequently, developing novel therapeutic strategies for NSCLC patients with BM is urgently needed. Cancer cells sensitive to *EGFR*-TKIs may retain their responsiveness to the targeted drug during tumor progression. Therefore, combining *EGFR*-TKIs with another treatment modality may inhibit the cancer sub-clones resistant to *EGFR*-TKIs. The FLAURA2 study demonstrated a longer median PFS in the osimertinib-chemotherapy group compared to the osimertinib group alone (24.9 months *vs*. 13.8 months) [37]. An ongoing phase III study, COMPEL (NCT04765059) [38], is comparing chemotherapy (pemetrexed plus platinum) plus osimertinib against chemotherapy plus placebo. Enhanced therapeutic outcomes are anticipated in the future. Additionally, combining *EGFR*-TKI with radiotherapy has been shown to prolong both CNS-PFS and OS in NSCLC patients with BM [39,40]. Further ongoing studies evaluate whether radiotherapy combined with *EGFR*-TKI is more effective than *EGFR*-TKI alone [41].

## Conclusions

Our study strongly suggests that BM is an independent predictor of poor prognosis for NSCLC patients with *EGFR* mutations receiving *EGFR*-TKIs, which may be attributed to a decline in peripheral lymphocytes. NSCLC patients with BM are more likely to experience metastases in other organs and have shorter survival times. Additionally, we identified a unique pattern of lymphatic infiltration with drainage vessels in the brain tumor tissues. These findings provide novel insights into the role of BM in the progression and prognosis of NSCLC and may inform future treatment strategies.

## Data Availability

Not applicable.
